# UPP1 Promotes Lung Adenocarcinoma Progression through Epigenetic Regulation of Glycolysis

**DOI:** 10.14336/AD.2022.0218

**Published:** 2022-10-01

**Authors:** Xuan Wang, Zheng Wang, Renhong Huang, Zhouyi Lu, Xiaofeng Chen, Dayu Huang

**Affiliations:** ^1^Department of Thoracic Surgery Huashan Hospital & Cancer Metastasis Institute, Fudan University, Shanghai 200040, China.; ^2^Department of General Surgery, Comprehensive Breast Health Center, Ruijin Hospital, Shanghai Jiao Tong University School of Medicine, Shanghai 200025, China

**Keywords:** Uridine phosphorylase 1, lung adenocarcinoma, glycolysis, epigenetic regulation, single-cell analysis

## Abstract

Uridine phosphorylase 1 (UPP1) is a dimeric enzyme that plays an indispensable role in pyrimidine salvage as well as uridine homeostasis and is upregulated in various cancers, including LUAD. However, the function and underlying mechanisms of UPP1 in mediating LUAD cell progression are still largely unknown. Single-cell RNA transcription analysis was applied to compare the expression of UPP1 in tumor tissues and adjacent tissue. In vitro gain- and loss-of-function experiments with LUAD cells were performed to elucidate the functions of UPP1. Western blotting, qRT-PCR, cell apoptosis, IHC staining, Seahorse XF24 Extracellular Flux analysis, chromatin immunoprecipitation (ChIP) assay, and bioinformatics analysis were performed to reveal the underlying mechanisms. In this study, UPP1 was found to be the top metabolism-related gene that was upregulated by single-cell transcriptomic profiling of LUAD. Next, we confirmed that UPP1 was highly expressed in LUAD tissues and cell lines and was correlated with poor overall survival in LUAD patients. UPP1 drove glycolytic metabolism and significantly regulated the sensitivity of tumors to glycolytic inhibitors in vitro and in vivo. UPP1 is subject to epigenetic regulation through histone acetylation. The CBP/p300 inhibitor SGC-CBP30 reduced the protein levels of UPP1, H3K27ac, and H3K9ac. ChIP assays revealed that acetyl-histone H3 and RNA polymerase II bind to the UPP1 promoter. UPP1 overexpression restored lactic acid production and glucose uptake compared to the SGC-CBP30 group. Our findings confirm UPP1 as a novel oncogene in LUAD, thus providing a potential novel diagnostic and therapeutic target for LUAD.

Lung cancer, one of the most pervasive human malignancies, remains the leading cause of cancer-associated death worldwide and imposes a substantial burden on both individuals and society [1]. Up to 80% of diagnosed lung cancer cases are non-small-cell lung cancer (NSCLC), of which lung adenocarcinoma (LUAD) remains the most common subtype [2, 3]. Although numerous studies have revealed that many genes, including metabolic regulating genes, play critical roles in LUAD progression, the pathogenesis and progression of this malignancy require further investigation [4, 5]. Tumors rely on a somatic evolutionary process in which deterministic tumor characteristics are preferentially selected for cell survival fitness in response to multiple external pressures, such as an adverse tumor microenvironment (TME) [6-8]. Of course, this also creates a dilemma in which it is difficult to effectively discriminate vital factors, thus creating barriers to the predictability of biomarker-based strategies based on biomarkers in precision therapy [9]. Consequently, cancer genomic diversity, especially intratumor heterogeneity (ITH), contributes to therapeutic failure, drug resistance, and ultimately lethal outcomes [10, 11]. Previous studies have indicated that at the single-cell level, molecular characterization of tumor cells might help reveal the temporal order of cancer cell initiation and cell evolutionary trajectories, enabling us to know more about the trajectory of cellular evolution [9, 12]. However, we still face a large number of challenges, and studies based on single-cell analysis need deep investigation to explain the exact theory of cancer evolution, which is needed for superior approaches to tumor intervention.

Cancer cells are able to undergo metabolic profile reprogramming to meet their requirements for proliferation, invasion and survival in hostile environments [13, 14]. Glucose is one of the key nutrients that not only plays an important role not only in energy production but also in tumorigenic metabolism and cancer progression [15]. During the process of oncogenesis, tumorigenic cells oxidize glucose by generating lactate and adenosine triphosphate (ATP) and even by modulating the Warburg effect [16]. The Warburg effect, characterized by enhanced aerobic glycolysis, high rates of lactate secretion and glucose uptake, is one of the essential pieces of metabolic reprogramming and a hallmark of cancer cells [17]. 2-Deoxy-D-glucose (2DG) is a stable glucose analogue that is actively taken up by the hexose transporters and phosphorylated by hydrogen, thus producing 2-DG-6P that could not be metabolized. It was indicated that cancer cells’ glucose metabolism could be targeted as a site for the intervention by agents such as 2-deoxy-D-glucose (2-DG) [18, 19]. Indeed, 2-DG exerts a vital role in extensive metabolic and biological processes, including cellular energy depletion, oxidative stress regulation, autophagy induction as well as inactivating signaling pathways like MAPK, PI3K/AKT, et al [20, 21]. Epigenetic regulation, including DNA methylation or histone acylation, can serve as a strategy to contribute to the Warburg effect [22-24]. In LUAD, the histone H2B monoubiquitination (H2Bub1)-mediated epigenetic pathway is closely correlated with the function of pyruvate kinase M2 (PKM2) in controlling the Warburg effect and tumorigenesis [25].

Uridine phosphorylase, characterized by its reversible phosphorolysis of uridine to uracil and ribose-1-phosphate, is considered as an indispensable enzyme in the pyrimidine salvage signaling pathway [26]. There are exist two isoforms of uridine phosphorylase, including UPP1 and UPP2. Compared with UPP2, UPP1 is widely distributed and extensively expressed. Uridine phosphorylase 1 (UPP1), encoded by the UPP1 gene, is a vital enzyme that is involved in uridine homeostasis and pyrimidine salvage. Emerging evidence indicates that the expression of UPP1 is associated with multiple malignant tumors, including colorectal cancer (CRC), breast cancer, pancreatic cancer, and thyroid carcinoma [27]. The expression of UUP1 is also reported to be associated with clinical significance and prognosis value [28, 29]. To the best of our knowledge, the role of UPP1 and its mechanisms in contributing to LUAD progression have not been explored. Thus, we enrolled and analyzed LUAD samples from the GSE131907 dataset, The Cancer Genome Atlas (TCGA) dataset and our own samples, aiming to characterize UPP1 expression and the uanderlying mechanism in LUAD both molecularly and clinically.

## MATERIALS AND METHODS

### Bioinformatics analysis

**Figure 1.**
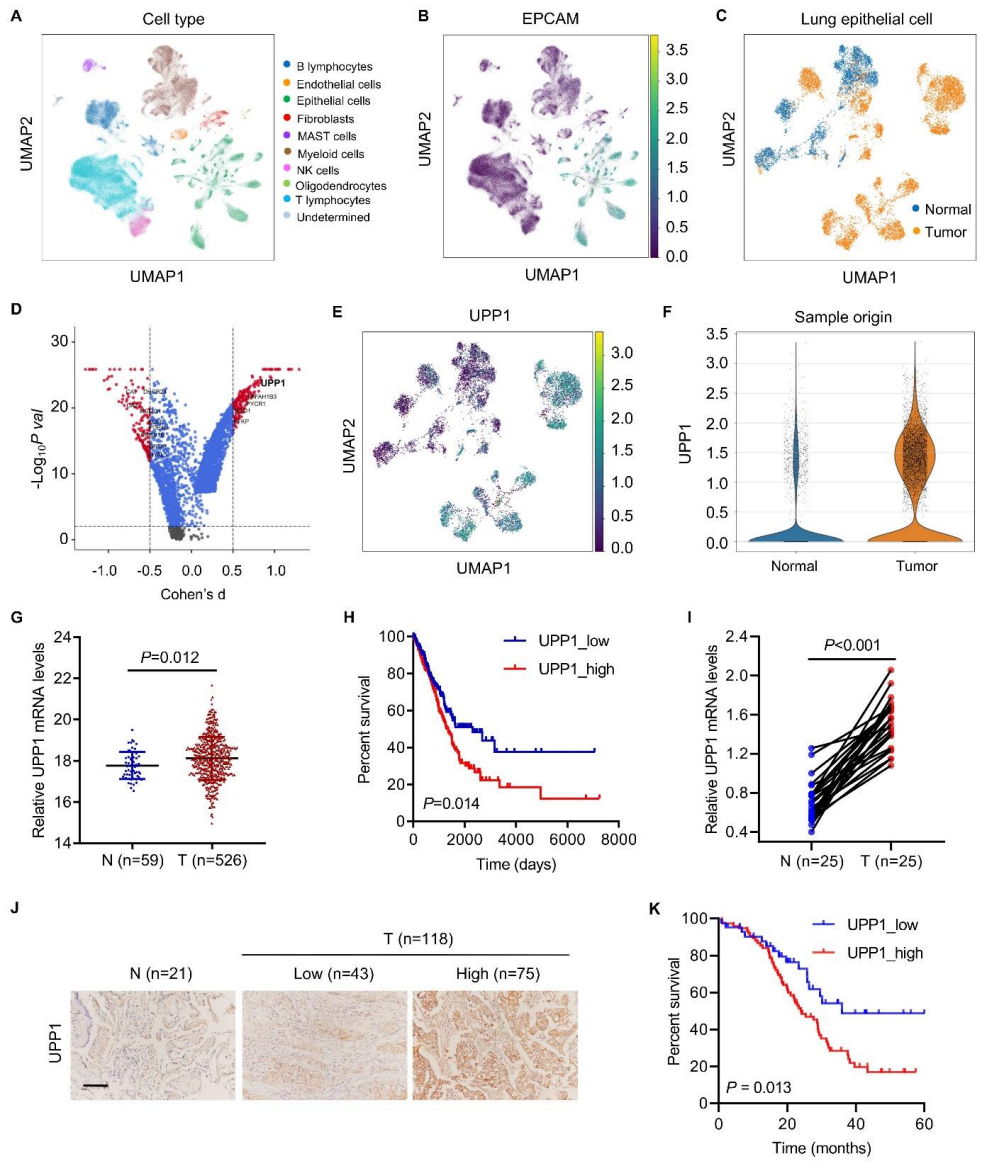
**UPP1 is the most upregulated metabolism-related gene and is associated with poor prognosis in lung adenocarcinoma**. (**A**) UMAP plot color-coded by variable clusters of LUAD in TCGA database. (**B**) UMAP plot, color-coded by the expression of EPCAM in lung epithelial cells. (**C**) UMAP plot of tumor and normal tissue transcriptomes. (**D**) Volcano plot showing differentially expressed genes between tumor cells and normal cells. The most significantly expressed gene, UPP1, is highlighted in bold. (**E**) UMAP plot, color-coded by expression of UPP1. (**F**) UPP1 expression between tumor and normal tissues. (**G**) mRNA expression of UPP1 in adjacent normal tissues (n=59) and tumor tissues (n=526) of LUAD in TCGA database. (**H**) UPP1 expression is associated with poor prognosis in TCGA database. (**I**) Real-time PCR analysis of UPP1 expression in paired LUAD adjacent normal tissues (n=25) and tumor tissues (n=25). (J-K) Immunohistochemical staining analysis showed that UPP1 is highly expressed in tumors and is associated with poor prognosis (normal tissues, n=21; UPP1 low-expression, n=43; UPP1 high-expression, n=75). Student's t test (Fig. 1G); non-parametric test (Fig. 1I); the Log-rank test (Fig. 1H, K).

We downloaded the single-cell RNA transcription data from the GSE131907 lung adenocarcinoma (LUAD) patient datasets in the Gene Expression Omnibus (GEO) database (www.ncbi.nlm.nih.gov/geo/) and the raw counts of the RNA-seq expression data from LUAD patient tissue samples from the TCGA database (http://cancergenome.nih.gov). We conducted normalization and then applied uniform manifold approximation and projection (UMAP), which is a technique well suited for visualization of high-dimensional data in a two-dimensional space, on the most variable genes across all cells to implement dimensionality reduction by projecting the original transcriptomic profiles to the eigenvector space (Fig. 1A). The gene set enrichment analysis (GSEA) algorithm was applied to identify pathways that were significantly enriched between the UPP1 high- and low-expression groups.

### Clinical samples

Between January 2018 and July 2020, twenty-five resected tumor samples and adjacent-normal samples were acquired from hospitalized patients with LUAD and frozen in liquid nitrogen before testing. The human LUAD tissue microarrays analyzed in this study were prepared by Shanghai Outdo Biotech Co., Ltd. (Shanghai, China) and collected for the detection of UPP1 protein expression by immunohistochemistry (IHC) analysis, and detailed information is provided in Table 1. All patients enrolled in the cohort signed the informed consent form. All experimental procedures followed the research protocols and present the retrieval method of cancer specimens and were approved by the Medical Ethics Committee of Huashan Hospital, Fudan University.

### Immunohistochemistry (IHC)

Paraffin-embedded tissue sections of LUAD and adjacent normal lung tissues were cut into 4-μm thick sections and used for immunohistochemical studies. IHC staining of UPP1 protein expression was performed on LUAD specimens following a standard protocol by incubation with rabbit polyclonal antibodies against human UPP1 antibody (Abcam; ab185680; dilution, 1:200) overnight, followed by incubation with goat monoclonal antibody against rabbit antibody (Shanghai Long Island Biotec. Co., Ltd; D-3004; dilution, 1:1000) for 1 h at room temperature. The immunohistochemical evaluation of UPP1 was performed and analyzed by two pathologists without knowledge of the clinical or pathological characteristics of these patients. The immunoreactivity was scored by two investigators using the H-score system, which ranged from 0-12, based on the percentage of positively stained cells (graded on a scale of 0-4: 0, <5%; 1, 5%-25%; 2, 25%-50%; 3, 50%-75%; 4, >75%) and the intensity of staining (graded on a scale of 0-3: 0, negative; 1, weak; 2, moderate; 3, strong). Based on the immunoreactivity scores, LUAD patients were categorized into low expression (H-score < 4) and high expression (H-score ≥ 4) groups.

### Cell culture and treatment

Human LUAD cell lines (H292, H358, H1299, H1975 and A549) and the bronchial epithelial cell line 16HBE were purchased from JRDUN Biotech (Shanghai, China). Cells were cultured in RPMI 1640 medium (HyClone, Logan, Invitrogen, Carlsbad, CA, USA) with 10% fetal bovine serum (Gibco, Life Technologies) in an incubator with an atmosphere of 5% CO_2_ and 37°C. Cells were treated with 25 mM glycolysis inhibitor 2-deoxygulcose glucose (2-DG) (Sigma-Aldrich, St. Louis, MO, USA), 5 μM CBP/p300 inhibitor SGC-CBP30 (MedChemExpress, New Jersey, USA) or vehicle.

### Gene overexpression and knockdown

To overexpress UPP1, the UPP1 gene was synthesized and cloned into the pcDNA3.1(+) vector (Addgene, Cambridge, MA, USA). Cell transfection was performed using Lipofectamine 2000 reagent (Invitrogen, Carlsbad, CA) according to the manufacturer’s instructions. Cells transfected with blank pcDNA3.1(+) vector were included as negative controls. To knockdown UPP1 expression by RNA interference, three shRNAs were synthesized and cloned into a pLKO.1 vector (Addgene, Cambridge, MA, USA). The short hairpin RNAs (shRNAs) used to target sites on the UPP1 gene and corresponding sequences were as follows: sh-1, 5ʹ-GCTGAAAGTCACAATGATT-3ʹ; sh-2, 5ʹ-CCGCTA TGCCATGTATAAA-3ʹ; and sh-3, 5ʹ-CCATGTGCAC CTTGGACTT-3ʹ. Recombinant plasmids and psPAX2 and pMD2G packaging vectors were applied to cotransfect 293T cells using Lipofectamine 2000 (Invitrogen, USA). Two days after transfection, the secreted virus particles were collected, concentrated by ultracentrifugation, and then transduced into cells. Viruses collected from cells transfected with pLKO.1-scramble shRNA (shNC) were included as negative controls.

### Cell viability assay

Cell viability was measured using the Cell Counting Kit-8 (CCK-8) (Dojindo, Japan) assay in accordance with the manufacturer’s instructions. Briefly, cells were seeded into 96-well plates at a density of 3×10^3^/well, followed by incubation with a 10 μL solution of CCK-8 with 5% CO_2_ at 37°C for 1 h. Cell proliferation was determined by the absorbance value (OD) at 450 nm using a microplate reader.

### Apoptosis assay

Cells were seeded into 6-well plates at a density of 5 × 10^5^ cells/well and allowed to grow until reaching 50% confluence. Flow cytometry was used to evaluate cellular apoptosis. Apoptosis was measured by propidium iodide and annexin-V staining. Briefly, the cells were incubated for 15 min in darkness at 4°C with 5 μL of FITC-labeled recombinant annexin V (annexin V-FITC), followed by incubation for another 15 min with 5 μL of propidium iodide. Apoptosis was profiled using a Beckman CytoFLEX Flow Cytometer (Beckman Coulter, Suzhou, China).

### Extracellular flux (XF) analysis

To measuring glycolysis and mitochondrial respiration, a Seahorse XF24 Extracellular Flux Analyzer was applied to monitor extracellular acidification rates (ECAR) and cellular oxygen consumption rates (OCR) in real time, as previously described [30].

### Measurement of glucose uptake

Glucose uptake was measured using a fluorescent glucose 2-deoxy-2-[(7-nitro-2,1,3-benzoxadiazol-4-yl (2-NBDG) glucose uptake assay kit (Biovision, Milpitas, CA, USA) according to the manufacturer’s protocol. In brief, 5×10^5^ cells per well in six-well plates were cultured at 37°C for 24 h and then starved of glucose for 3 h. After incubating with Krebs-Ringer bicarbonate buffer supplemented with 2% bovine serum albumin (BSA) for 40 min, 2-NBDG (100 µM) was added to each well and incubated for 45 min at 37°C. Subsequently, the cells were washed with PBS three times, trypsinized, and then resuspended in 10% FBS before flow cytometry analysis. The potential glucose uptake of the samples was measured using a BD Accuri C6 flow cytometer (BD Biosciences, Franklin Lakes, NJ, USA).

### Measurement of lactate

Cells (5×10^5^ cell/well) were grown in six-well plates and maintained for one day at 37°C. After two days of treatment, the lactate released from the cells was measured using a lactic acid assay kit (Nanjing Jiancheng Bioengineering Institute, China) according to the manufacturer’s instructions.

### Chromatin immunoprecipitation (ChIP) assay

The SimpleChIP® Plus Enzymatic Chromatin IP Kit (Magnetic Beads) (Cell Signaling Technology, Danvers, MA, USA; #9005S) was used to perform the ChIP-seq experiment. Cells were fixed in 1% formaldehyde, harvested, sonicated, and then incubated with anti-acetyl-histone H3 (AcH3; Upstate Biotechnology, Los Altos, CA, USA; 06-599), anti-RNA polymerase II (RNA pol II; Covance, MMS-126R), or IgG antibody (Cell Signaling Technology, #2729). Purified ChIP DNA was confirmed by PCR using primers for the UPP1 promoter (F, 5’-GG CTTGTCTGCGGGATG-3’ and R, 5’-CGGAGCACTC GAATGAGG-3’).

### Quantitative RT-PCR (RT-qPCR)

Total RNA was purified using TRIzol Reagents (Invitrogen) and reverse transcribed into cDNA with the PrimeScript kit (Thermo Fisher) in accordance with the protocol. RT-qPCR was then performed with the SYBR® Green Kit (Thermo Fisher) in an ABI 7300 Real-Time PCR System (Applied Biosystem, USA). GAPDH was used as an internal control. The primer sequences were as follows: UPP1-F: 5ʹ-ATGGGCATTCCTTCTATC-3ʹ, UPP1-R: 5ʹ-GACAATCTGCTCAAACTC-3ʹ; GAPDH-F: 5ʹ-AATCCCATCACCATCTTC-3ʹ, GAPDH-R: 5ʹ-AGGCTGTTGTCATACTTC-3ʹ. The expression level of the target mRNA was normalized by the expression of GAPDH and β-actin. Relative mRNA expression was calculated using the 2^-ΔΔCT^ method.

### Western blot

Whole-cell lysates were extracted using radio-immunoprecipitation assay (RIPA) lysis buffer supplemented with protease inhibitor cocktail (Sigma, USA), and the proteins were separated by SDS-PAGE, followed by transfer onto nitrocellulose membranes (Millipore, Bedford, USA). The membranes were then blocked and incubated with individual detection antibodies against UPP1 (Abcam; ab128854), ENO1 (Abcam; ab155102), LDHA (Invitrogen; PA5-23036), H3K27ac (Abcam; ab45173), H3K9ac (Abcam; ab10812), and GAPDH (Proteintech; 60004-1-1G) at 4°C overnight. After washing with TBST three times for 5 min each, the membranes were probed with HRP-conjugated secondary antibody (Beyotime, Shanghai, China; A0208, A0216) at room temperature for 1 h. Finally, the membranes were visualized using an enhanced chemiluminescence system (Bio-Rad, USA). GAPDH was employed as an internal control.

### In vivo tumor xenograft model

Four- to six-week-old male nude mice were purchased from the Shanghai Laboratory Animal Company. All experimental procedures were performed in accordance with the animal ethics guidelines and protocols approved by the Medical Ethics Committee of Huashan Hospital, Fudan University. A total of 5×10^6^ H1975 cells transduced with shNC or UPP1 shRNA vector (sh-1) were subcutaneously injected into the armpits of mice (n=6 per group). Otherwise, mice were subcutaneously injected in the armpits with H1975 or H1299 cells (5×10^6^), and starting on day 12, each mouse received an intraperitoneal injection of 250 mg/kg/d 2-DG (Sigma-Aldrich) or vehicle every other day (n=6 per group). Measurements of tumor size and volume were recorded and calculated every three days. After 21 days, the mice were euthanized, tumor xenografts were collected, imaged, and weighed, and deoxynucleotidyl transferase dUTP nick end labeling (TUNEL) (Roche, Indianapolis, IN, USA) analysis was performed. Bioluminescence imaging was conducted using an IVIS 200 imaging system coupled to a data acquisition computer running Living Image Software (XENOGEN).

### Statistical analysis

All experiments in the current study were independently conducted at least three times. Data are shown as the mean value ± SD. The statistical analyses were conducted using GraphPad Prism 8.0.2. Whether the data were normally distributed was tested using the Shapiro-Wilk normality test. The measurement data between two groups were compared using the Student's t test if they were normally distributed. One-way analysis of variance (ANOVA) test was performed among three or more groups if the variation was comparable. If the data were not normally distributed, comparisons were performed by non-parametric tests. The overall survival rate was calculated according to the Kaplan-Meier method and Cox’s proportional hazards regression model. The log-rank test was used to calculate the significant difference between groups. *P*<0.05 was considered to be statistically significant.

**Figure 2.**
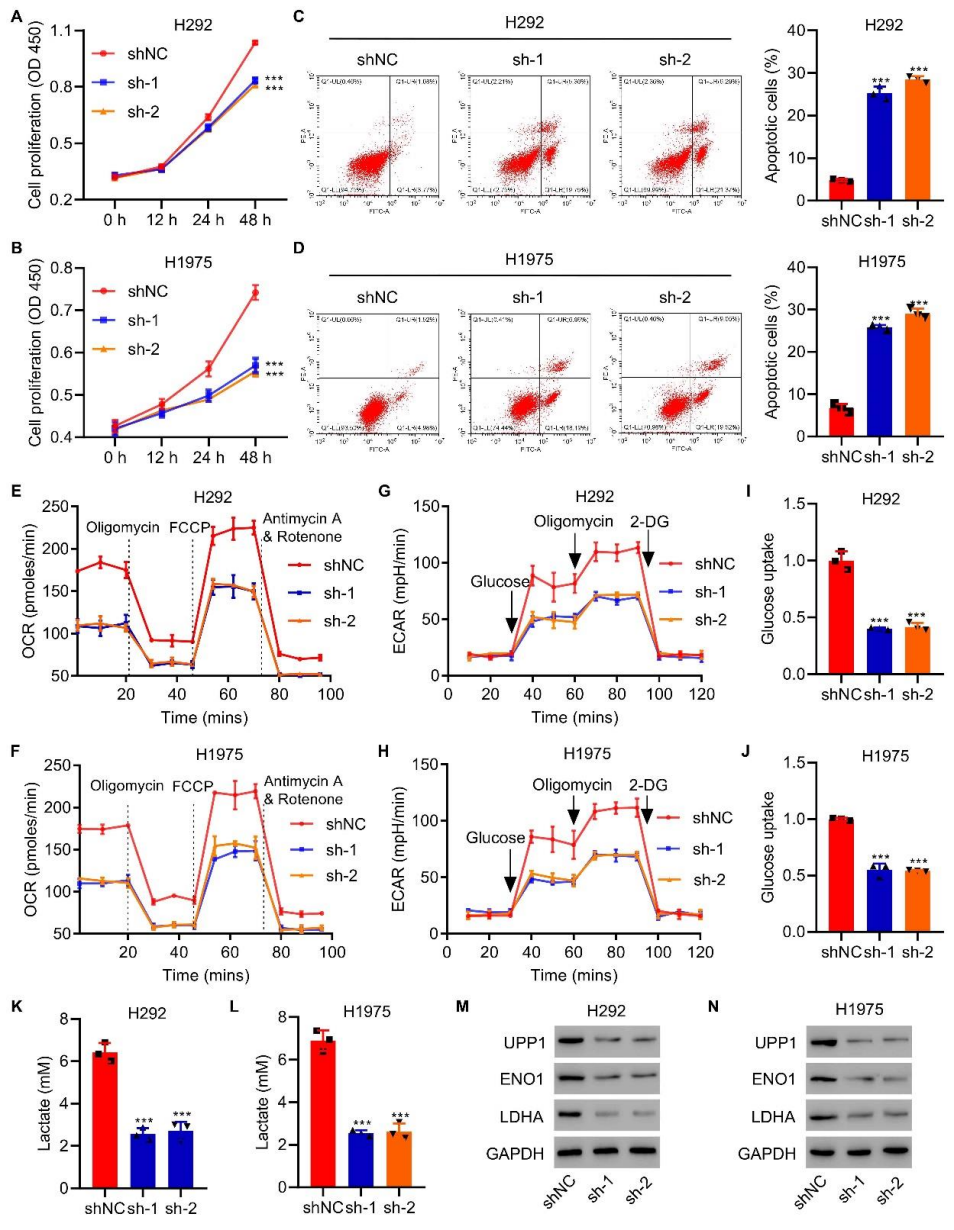
**UPP1 drives glycolytic metabolism in LUAD**. (A-D) UPP1 knockdown in H1975/H292 cells suppressed cell proliferation and promoted apoptosis. (E-H) UPP1 knockdown significantly reduced OCR and ECAR levels in H292 and H1975 cells compared to control cells. (I-L) Lactic acid production and glucose uptake were significantly decreased in response to UPP1 knockdown in H292 and H1975 cells. (M-N) western blot analysis showed that UPP1 inhibits the expression of ENO1 and LDHA. One-way ANOVA in (Fig.2A-D, I-L). ***P<0.001 vs. shNC.

**Table 1:** Clinicopathological features of LUAD patients and UPP1 expression.

Clinicopathological parameter	UPP1		P value
	Low group, No. of patients	High group, No. of patients	
**Age**			0.072
≤60	24	29	
>60	19	46	
**Sex**			0.637
Female	17	33	
Male	26	42	
**Tumor size (cm)**			0.002
≤4	31	32	
>4	12	43	
**Smoking status**			0.006
Never	28	29	
Former and current smokers	15	46	
**TNM stage**			0.032
I-II	26	30	
III-IV	17	45	
**Lymph node metastasis**			0.031
N0	30	37	
N1-3	13	38	
**Distant metastases**			0.013
M0	28	31	
M1	15	44	
**Recurrence**			0.002
No	33	36	
Yes	10	39	

P < 0.05 represents statistical significance (Chi-square test).

## RESULTS

### UPP1 is the top metabolism-related gene that is upregulated in single-cell transcriptomic profiling of lung adenocarcinoma

To examine the biodiversity of the tumor cell community, we obtained single-cell RNA transcription data from the GSE131907 LUAD patient datasets. These cells were grouped into ten clusters, primarily according to their cell types, as annotated based on known cell lineage-specific marker genes unique to T lymphocytes, B lymphocytes, fibroblasts, NK cells, mast cells, myeloid cells, epithelial cells, endothelial cells, oligodendrocytes, and undetermined cells. The analyses revealed that some cases formed distinct clusters, while others overlapped with each other. To investigate the genes specifically expressed in LUAD tumor tissue, we extracted lung epithelial cells from the single-cell dataset (Fig. 1B). To determine whether the genes exert differential effects on LUAD tumorigenesis, we compared the scRNA-seq transcriptome of each lung epithelial cell between tumor tissue and normal tissue (Fig. 1C). Among the differentially expressed transcripts, UPP1 was most significantly expressed in the tumor tissue (Fig. 1D). UMAP analysis showed that UPP1 was primarily enriched in lung epithelial cells (Fig. 1E), especially in LUAD cells (Fig. 1F). We found that the expression of UPP1 in tumor tissues was significantly correlated with tumor size, smoking status, TNM stage, lymph node metastasis, distant metastases, and recurrence (Table 1).

### UPP1 is highly expressed in LUAD and is associated with poor prognosis

In the TCGA database, UPP1 was prominently upregulated in tumors compared to normal tissues when analyzing the transcription profiles of 526 LUAD tumors and 59 corresponding nontumorous tissues (Fig. 1G). Upregulated expression of UPP1 was notably associated with poor overall survival (OS) (Fig. 1H). Consistently, RT-PCR analysis of UPP1 expression in paired LUAD tumor tissues (n=25) and adjacent normal tissues (n=25) revealed that UPP1 was upregulated (Fig. 1I). Furthermore, immunohistochemical staining analysis showed that UPP1 was highly expressed in tumors (Fig. 1J) and was associated with poor prognosis (normal tissues, n=21; UPP1 low-expression, n=43; UPP1 high-expression, n=75) (Fig. 1K). We found that patients with stage IV have a higher UPP1 expression level than those with stage III (Supplementary Fig. 6). Western blot also made a validation in fresh clinical samples that UPP1 was highly expressed in tumors (Supplementary Fig. 9). All the above evidence indicates that UPP1 is involved in the progression of LUAD and is a critical diagnostic, prognostic and predictive biomarker in lung cancer.

**Figure 3.**
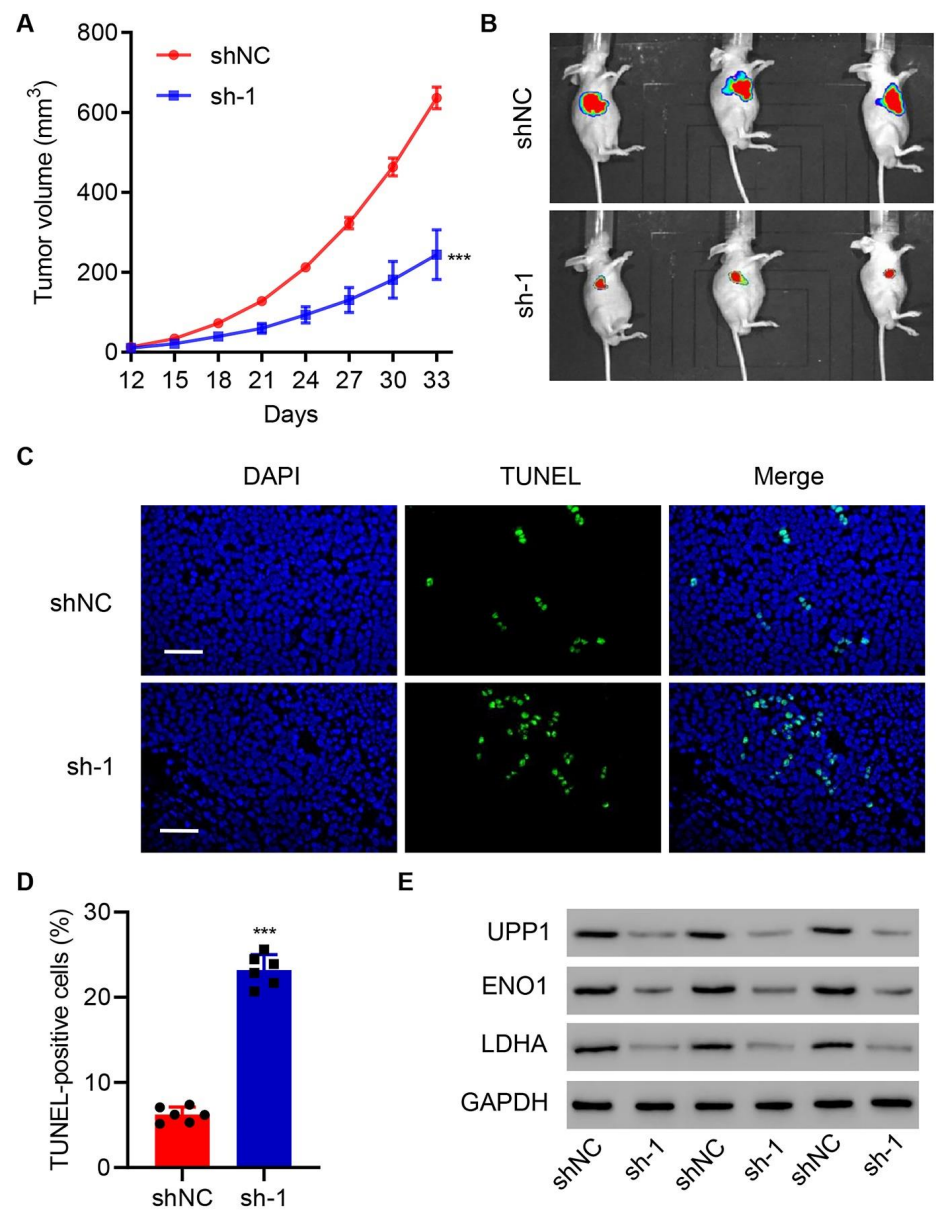
**UPP1 knockdown suppresses tumor growth in vivo**. (**A-B**) UPP1 knockdown suppresses subcutaneous tumor growth in nude mice (shNC group, n=3; sh-UPP1, n=3). The tumor volumes in H1975 cells transfected with shUPP1 and control H1975 cells and used for subcutaneous models are shown. (**C-D**) UPP1 knockdown promotes apoptosis of tumor cells. (**E**) UPP1 knockdown inhibits the protein expression of ENO1 and LDHA. Student's t test (Fig. 3A, D). The experiments were independently conducted six times. **P<0.01, ***P<0.001 vs. shNC. Scale bar: 50 μm.

**Figure 4.**
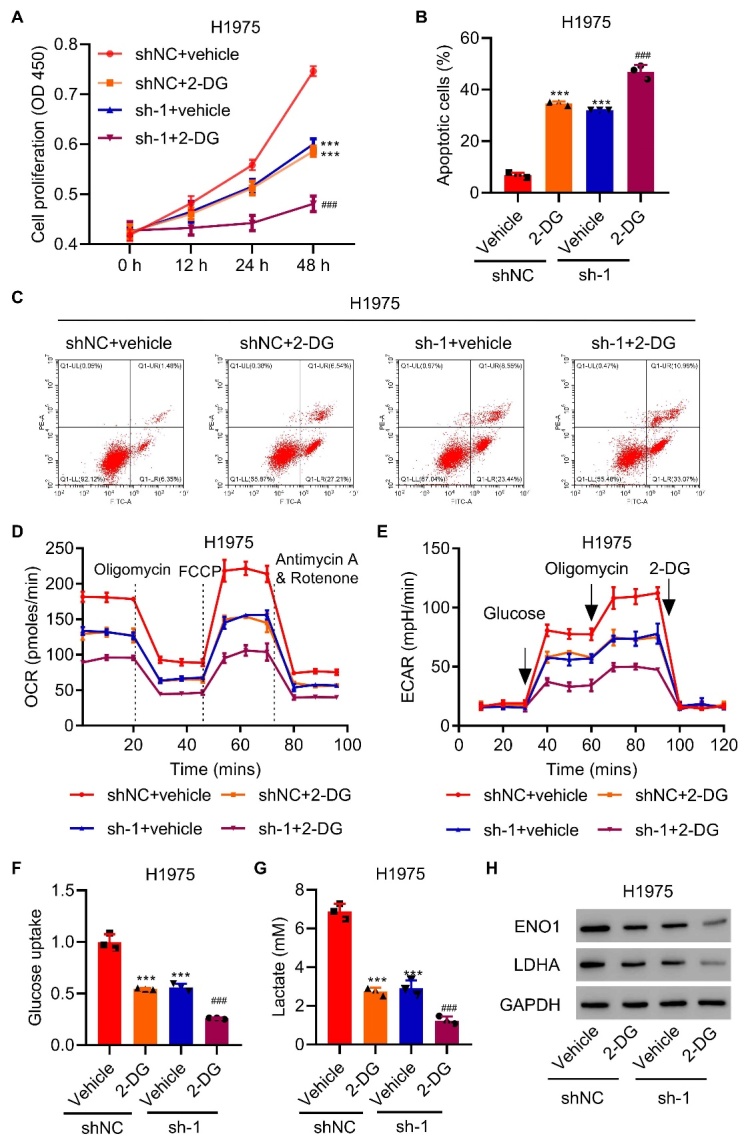
**UPP1 knockdown increases the antitumor effect induced by 2-DG**. (**A**) UPP1 knockdown enhances the inhibitory effects of 2-DG on cell proliferation. (B-C) UPP1 knockdown reinforces the degree of apoptosis induced by 2-DG. (D-E) UPP1 knockdown significantly decreases OCR and ECAR levels compared to the 2-DG group. (F-G) UPP1 knockdown significantly reduces lactic acid production and glucose uptake in H1975 cells. (**H**) Expression of ENO1 and LDHA in UPP1 knockdown cells with 2-DG, as determined by western blot. One-way ANOVA (Fig. 4A, B, F, G). The experiments were independently conducted three times. **P<0.01, ***P<0.001 vs. shNC+vehicle. ^###^P<0.001 vs. shNC+2-DG.

**Figure 5.**
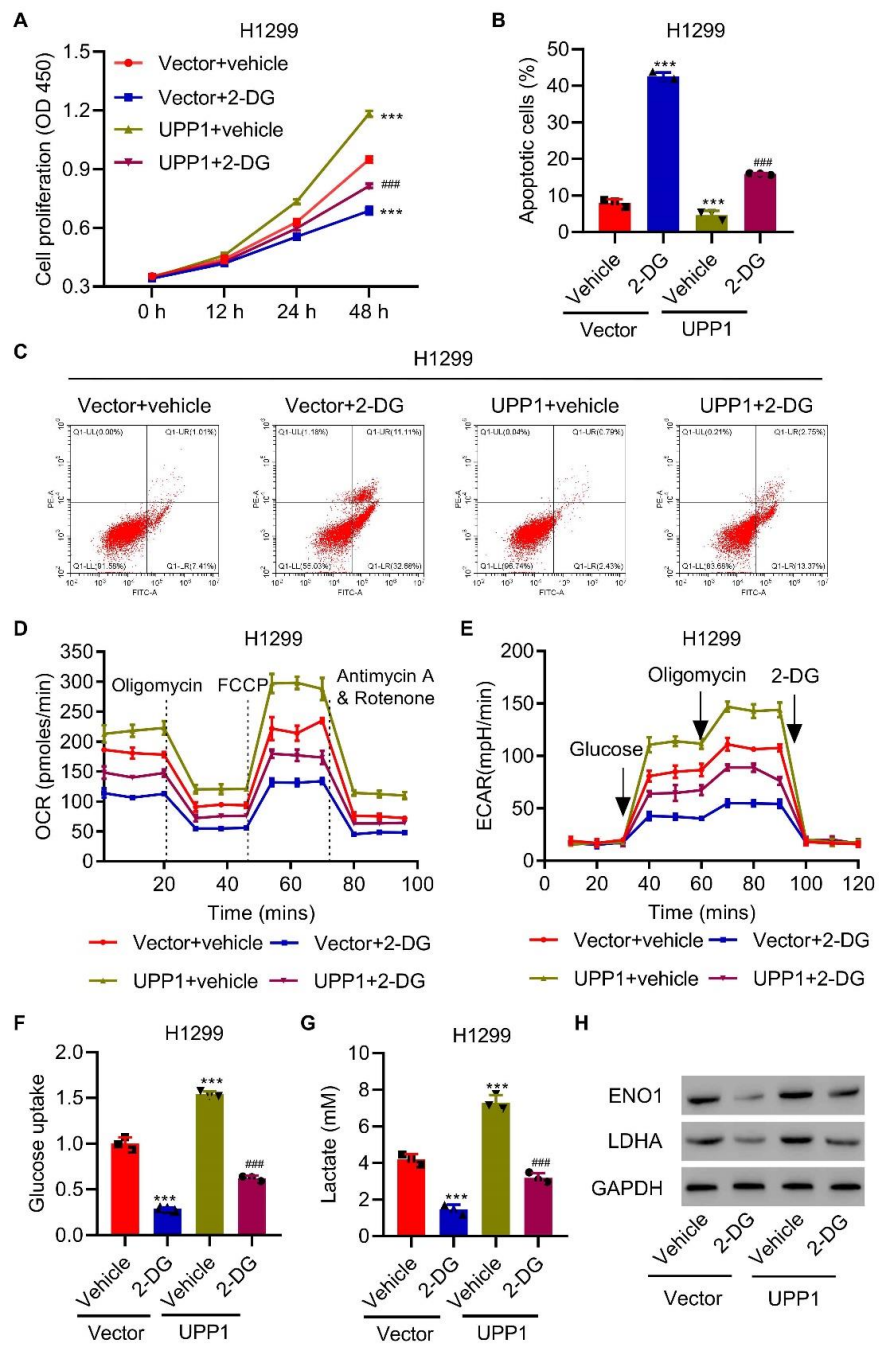
**UPP1 overexpression reduces the antitumor effect induced by 2-DG**. (**A**) UPP1 overexpression in H1299 cells alleviates the inhibition of cell proliferation by 2-DG. (B-C) UPP1 overexpression reduced apoptosis and even reversed apoptosis. (D-E) UPP1 overexpression reinstated OCR and ECAR levels compared to the 2-DG group. (F-G) UPP1 overexpression reinstated lactic acid production and glucose uptake. (**H**) Expression of ENO1 and LDHA in UPP1-overexpressing cells with 2-DG, as determined by western blot. One-way ANOVA (Fig. 5A, B, F, G). The experiments were independently conducted three times. **P<0.01, ***P<0.001 vs. vector+vehicle. ^###^P<0.001 vs. vector+2-DG.

### UPP1 drives glycolytic metabolism in LUAD

To further demonstrate the features of UPP1 in LUAD, we used GSEA to explore relevant pathways. The results showed that the expression of UPP1 is related to tumor glycolysis and the apoptosis pathway (Supplementary Fig. 2A-B). To analyze the role of UPP1, we measured the expression of UPP1 in different LUAD cell lines and found that UPP1 was relatively more highly expressed in H292 and H1975 cell lines by qRT-PCR and western blot analysis and found that UPP1 was more highly expressed in the H292 and H1975 cell lines (Supplementary Fig. 1A). In addition, we also established lentiviral-mediated stable UPP1-silenced H292 and H1975 cell lines with three different shRNA sequences and verified by qRT-PCR and western blot that that UPP1 expression could be specifically decreased (Supplementary Fig. 1B-C). We next examined the function of UPP1 in LUAD cells and found that knockdown of UPP1 dramatically suppressed cell proliferation (Fig. 2A-B) and promoted apoptosis (Fig. 2C-D) in H292 and H1975 cells. Knockdown UPP1 induced by siRNA could effectively inhibit the proliferation of H292 and H1975 cell lines (Supplementary Fig. 4A-B). In addition, the proliferation effect induced by UPP1 knockdown could be reversed by the UPP1 rescue (Supplementary Fig. 5A-B).

To determine whether alteration of UPP1 directly influences glycolytic metabolism, we measured the oxygen consumption rate (OCR) and extracellular acidification rate (ECAR) in LUAD cells after manipulating UPP1. Knockdown of UPP1 significantly reduced OCR and ECAR levels in H292 and H1975 cells (Fig. 2E-H) compared to control cells. In addition, the mRNA expression of UPP1 was positively associated with the mRNA expression of ENO1 (Supplementary Fig. 3A) and LDHA (Supplementary Fig. 3B).

Further functional colorimetric validation showed that lactic acid production, a key metabolite of glycolysis, and glucose uptake were both significantly decreased in response to UPP1 knockdown in H292 cells and H1975 cells (Fig. 2I-L). Furthermore, the protein expression of glycolytic genes was verified by western blot, and ENO1 and LDHA were much lower in cells with UPP1 knockdown (Fig. 2M-N). UPP1 overexpression increases the cell proliferation, and protein expression of ENO1 and LDHA in vivo (Supplementary Fig. 7A-C).

To evaluate the effect of UPP1 in vivo, H1975 cells with stable UPP1 knockdown were subcutaneously implanted into nude mice, and tumor size was monitored every three days. UPP1 Knockdown group (n=3) suppressed tumor growth and reduced tumor size compared to the control group (n=3) (Fig. 3A-B). In-depth analysis of the tumor revealed that UPP1 Knockdown induced apoptosis (Fig. 3C-D) and decreased the expression of ENO1 and LDHA (Fig. 3E). Taken together, this evidence suggests that UPP1 plays a pivotal role in promoting LUAD progression.

**Figure 6.**
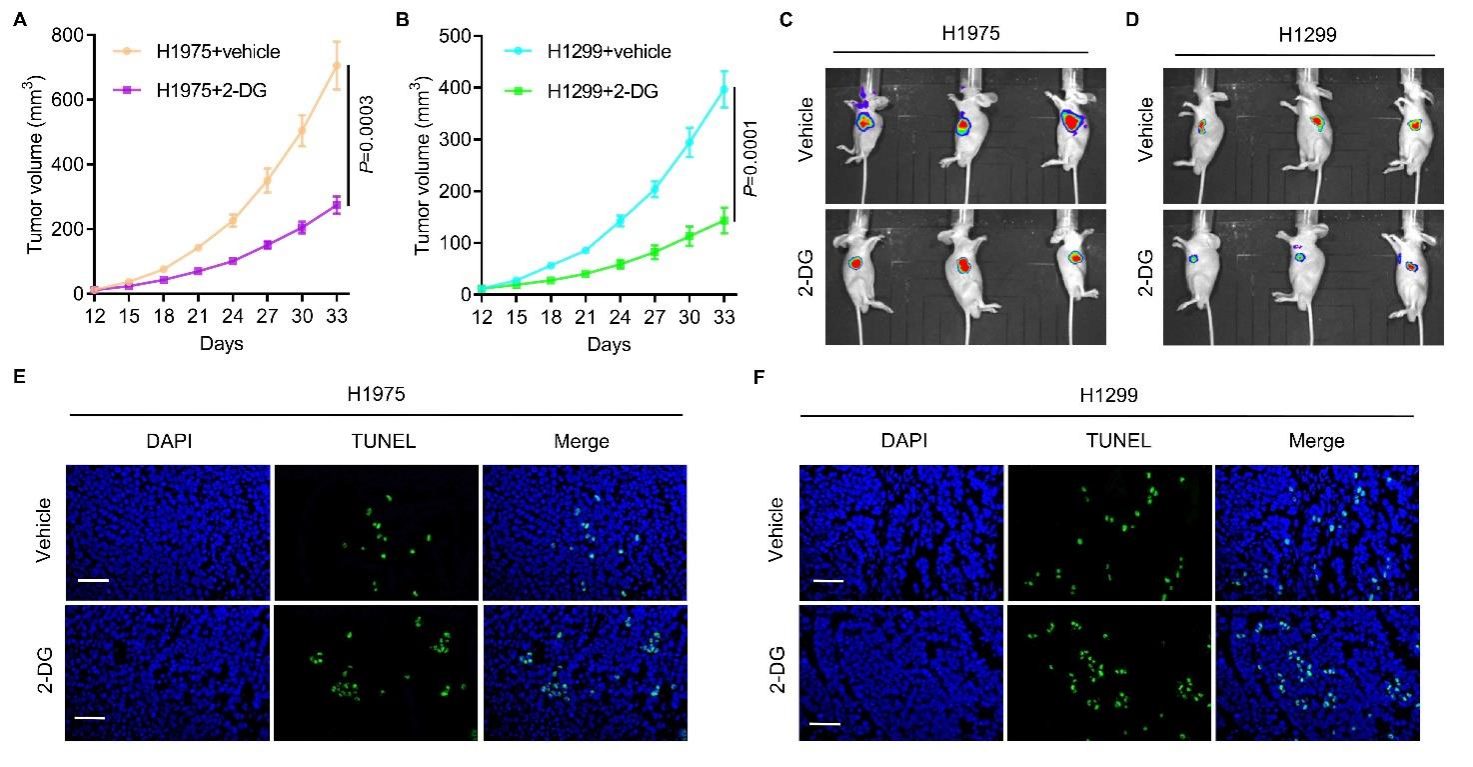
**UPP1 significantly alters the sensitivity of tumors to glycolytic inhibitors in vivo**. (A-D) 2-DG effectively suppresses subcutaneous tumor growth in nude mice (Vehicle group, n=3; 2-DG group, n=3). The tumor volumes in H1975+vehicle and H1975+2-DG, H1299+vehicle and H1299+2-DG in subcutaneous models are shown. (E-F) 2-DG effectively promotes apoptosis of tumor cells. H1299 cells with low UPP1 expression were more sensitive to 2-DG than H1975 cells with high UPP1 expression. One-way ANOVA in (Fig. 6A-B). Scale bar: 50 μm

**Figure 7.**
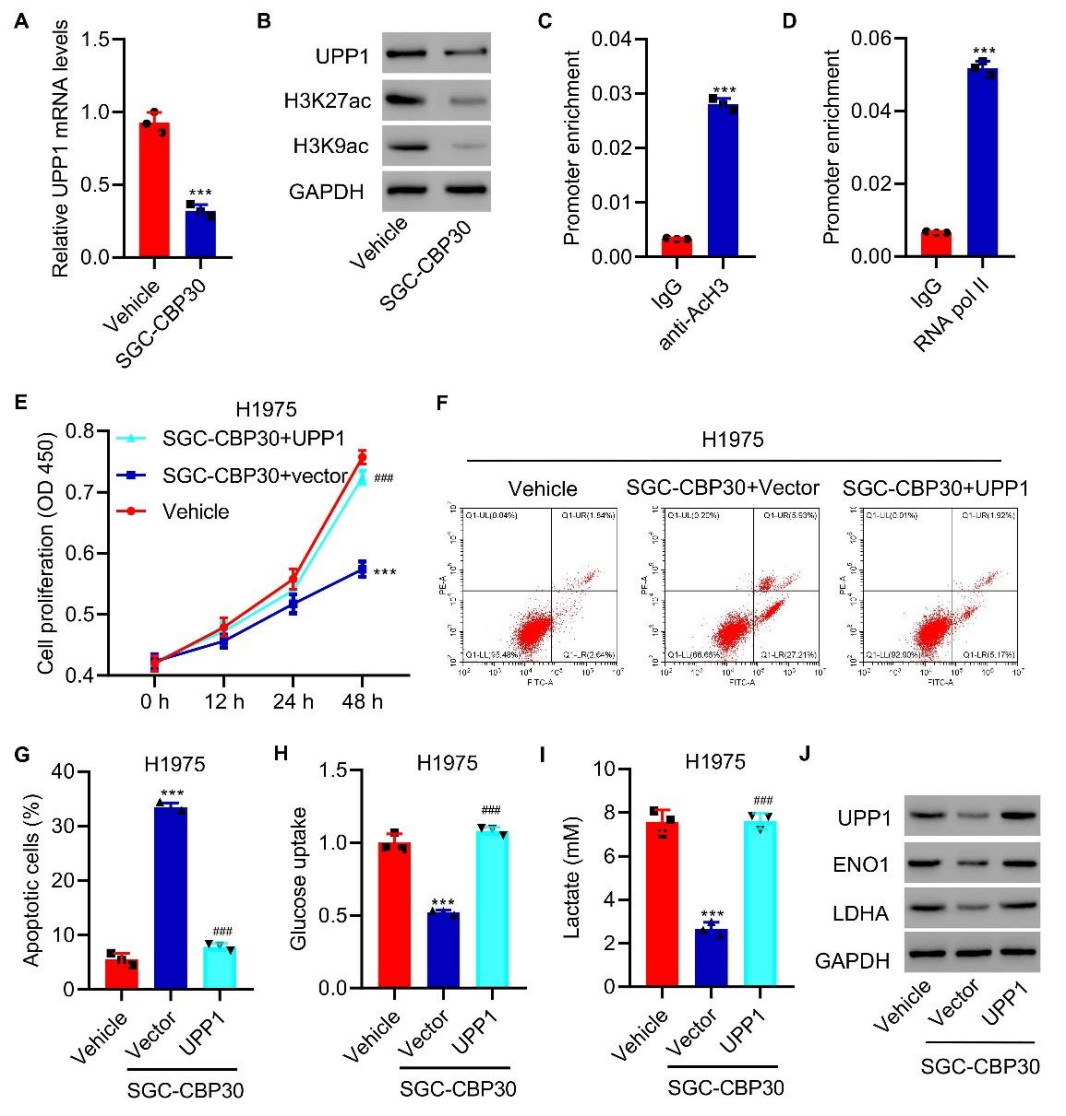
**UPP1 overexpression inhibits the antitumor effect induced by SGC-CBP30**. (**A**) Expression of UPP1 in H1975 cells treated with SGC-CBP30. (**B**) Protein expression of H3K27ac and H3K9ac in H1975 cells treated with SGC-CBP30. (C-D) ChIP assay showing that acetyl-histone H3 and RNA polymerase II binds to the UPP1 promoter. (**E**) UPP1 overexpression decreases the inhibitory effects on cell proliferation in response to SGC-CBP30. (F-G) UPP1 overexpression reduces the degree of apoptosis induced by SGC-CBP30. (H-I) UPP1 overexpression increases lactic acid production and glucose uptake in H1975 cells. (**J**) Expression of ENO1 and LDHA in UPP1-overexpressing SGC-CBP30 cells compared to control cells, as determined by western blotting. Student t test in (Fig. 7A, C-D); One-way ANOVA (Fig. 7E, G-I). The experiments were independently conducted three times. ***P<0.001 vs. vehicle or IgG. ^###^P<0.001 vs. SGC-CBP30+vector.

### UPP1 regulates the antitumor effect induced by the glycolytic inhibitor 2-DG

To explore the relationship between UPP1 and glycolytic metabolism, we used a glycolytic inhibitor, 2-deoxy-D-glucose (2-DG), in this study. Adding 2-DG markedly inhibited H1975 cell proliferation. Moreover, UPP1 knockdown enhanced the inhibitory effects of 2-DG on cell proliferation (Fig. 4A). Additionally, UPP1 knockdown reinforced the degree of apoptosis induced by 2-DG (Fig. 4B-C).

To confirm the variation in UPP1-mediated glycolytic metabolism with or without 2-DG, we found that 2-DG reduced OCR and ECAR levels in H1975 cells compared to control cells. UPP1 knockdown significantly decreased OCR and ECAR levels compared to those in the 2-DG group (Fig. 4D-E). UPP1 knockdown also significantly reduced lactic acid production and glucose uptake in H1975 cells, even after 2-DG had already reduced them (Fig. 4F-G). Additionally, ENO1 and LDHA were even lower in UPP1 knockdown cells with 2-DG, as shown by western blot analysis (Fig. 4H).

To confirm the role of UPP1, we overexpressed UPP1 in H1299 cells and verified it by qRT-PCR and western blot (Supplementary Fig. 1D). In contrast to the effects of knockdown, we found that UPP1 overexpression in H1299 cells alleviated the inhibition of cell proliferation by 2-DG (Fig. 5A). The UPP1 overexpression group exhibited reduced apoptosis and even reversed the levels of apoptosis (Fig. 5B-C). UPP1 overexpression restored OCR and ECAR levels compared to the 2-DG group (Fig. 5D-E), as well as lactic acid production and glucose uptake (Fig. 5F-G). The protein expression of ENO1 and LDHA was lower in the 2-DG group, but UPP1 overexpression restored the expression (Fig. 5H).

To evaluate these effects in vivo, 2-DG was applied to H1975 and H1299 cells subcutaneously implanted in nude mice. 2-DG suppressed tumor growth (Fig. 6A-D) and promoted apoptosis (Fig. 6E-F). H1299 cells with lower UPP1 expression exhibited enhanced sensitivity to 2-DG compared to H1975 cells with higher expression of UPP1 (Fig. 6A-F). These data reveal that UPP1 significantly changes the sensitivity of tumors to glycolytic inhibitors both in vitro and in vivo.

### UPP1 is subject to epigenetic regulation through histone acetylation

To further examine the role of UPP1 in LUAD, we found that the CBP/p300 inhibitor SGC-CBP30 reduced the expression of UPP1 (Fig. 7A), H3K27ac and H3K9ac (Fig. 7B). ChIP assays showed that acetyl-histone H3 and RNA polymerase II could bind to the UPP1 promoter (Fig. 7C-D). SGC-CBP30 inhibited cell proliferation and induced apoptosis, while UPP1 overexpression restored cell proliferation in vitro (Fig. 7E) and in vivo (Supplementary Fig. 8A-B), and inhibited apoptosis (Fig. 7F-G). UPP1 overexpression restored lactic acid production and glucose uptake compared to the SGC-CBP30 group in vitro (Fig. 7H-I) and in vivo (Supplementary Fig. 8C). The protein expression of ENO1 and LDHA was reduced in the SGC-CBP30 group, but UPP1 overexpression increased their expression (Fig. 7J).

## DISCUSSION

Molecular and intratumor heterogeneity remain a key challenge for the highly precise diagnosis and efficient therapy of oncologic patients [31]. LUAD is a malignant tumor that is associated with a high level of molecular heterogeneity with various mechanisms of origin, including genetic, epigenetic and nongenetic sources [32, 33]. Recent advances in single-cell genomic technologies have provided an unprecedented amount of data on the profile of RNA, protein, and chromatin states of cells within tissues [34]. These advances have dramatically improved the way we understand collective behavior and regulatory mechanisms within a tumor ecosystem [35]. In this study, we analyzed single-cell data from the TCGA database to identify metabolism-related genes with statistically significant differences. UPP1 was the top metabolism-related gene upregulated in the single-cell transcriptomic profiling of LUAD. UPP1 was also more highly expressed in tumorous tissues than in normal tissues, and higher expression of UPP1 was associated with worse survival outcomes. Furthermore, UPP1 promotes glycolytic metabolism and tumor growth and inhibits the antitumor effect induced by epigenetic regulation through histone acetylation. To the best of our knowledge, our study is the first to investigate the mechanism of UPP1 and to reveal how UPP1 exerts antitumor effects by epigenetic regulation in LUAD.

Indeed, UPP1 overexpression has been observed, and UPP1 is reported to be an oncogene that is related to cancer progression and prognosis [36]. Wang J et al. demonstrated that UPP1 was prominently expressed in mesenchymal subtypes of brain glioma and exerted an oncogenic role by suppressing the tumor-associated immune response [27]. In thyroid cancer, UPP1 was elevated in carcinoma tissue compared to adjacent tissue and was markedly associated with lymph node involvement [28]. In LUAD, GSEA demonstrated that tumor glycolysis and apoptosis pathways in cancer are significantly enriched in response to UPP1 alteration in LUAD. The bioinformatics analysis has been functionally validated in several in vitro and in vivo experimental models. In cultured LUAD cells and xenograft mouse models, UPP1 downregulation markedly suppresses tumor growth and inhibits glycolysis progression in LUAD. Furthermore, we found that UPP1 significantly altered the sensitivity of tumors to glycolytic inhibitors. Previous studies are consistent with ours in indicating that UPP1 might serve as an oncogene that regulates tumor progression by the regulation of glycolysis. UPP1 performs a function of supporting the process like glycolysis, the Krebs cycle (also known as tricarboxylic acid cycle), nucleotide metabolism, et al [37].

The Warburg effect, which originally described increased production of lactate in cancer, is associated with diverse cellular processes, such as angiogenesis, hypoxia, macrophages polarization and T-cell activation [38-40]. Alterations in tumor-associated metabolism contribute to the maintenance and establishment of a tumorigenic state [41]. The Warburg effect refers to tumor cells taking in glucose to produce a large amount of lactate, thus manufacturing hydrochloric acid, and favoring processes such as metastasis, angiogenesis and immunosuppression [42]. Moreover, the Warburg effect involves key genes, including glucose transporter 1 (GLUT1), hexokinase 2 (HK2), enolase 1 (ENO1), pyruvate kinase M2 (PKM2) and lactate dehydrogenase A (LDHA), as well as alterations in metabolic enzymes, such as GLUT1 and LDHA. To the best of our knowledge, the relationship between UPP1 and the Warburg effect has not been investigated. In our study, we observed that lactic acid production and glucose uptake were significantly decreased in response to UPP1 knockdown, and UPP1 knockdown increased the antitumor effect induced by 2-DG, suggesting that that UPP1 is capable of regulating tumor progression through the Warburg effect.

Previous studies have reported that CBP/p300 epigenetically regulates the expression of glycolysis-related metabolic enzymes through the modulation of histone acetylation in HCC and highlights the value of targeting the histone acetyltransferase activity of CBP/p300 for HCC therapy [43]. In our study, UPP1 was subject to epigenetic regulation through histone acetylation. The CBP/p300 inhibitor SGC-CBP30 reduced the expression of UPP1 H3K27ac and H3K9ac, as shown by western blotting. ChIP assays revealed that acetyl-histone H3 and RNA polymerase II could bind to the UPP1 promoter. UPP1 Overexpression restored lactic acid production and glucose uptake compared to the SGC-CBP30 group. All the evidence shows that UPP1 is subject to epigenetic regulation by histone acetylation.

Finally, how UPP1 contributes to LUAD progression and the mechanism through which it influences the antitumor effects of 2-DG in LUAD, have yet to be elucidated. It will be interesting to see whether UPP1 affects the inhibition of thymidylate synthase, which has been suggested to influence antitumor drug responses. Understanding the functions and mechanism of UPP1 in homeostasis is also critical for assessing the global effects of interfering with its expression in LUAD. Whether there are other downstream targets and mediators of UPP1 in LUAD and, if so, their precise contribution also needs to be investigated in the future work.

Our findings might be the first to show the biological function of UPP1, which was selected using single-cell transcriptomic analysis in LUAD, revealing a reasonable mechanism by which UPP1 contributes to LUAD proliferation through the epigenetic regulation of glycolysis. Our findings provide novel insight into the biological function of UPP1 in LUAD, and UPP1 may represent a potential therapeutic target for LUAD.

## Supplementary Materials

The Supplementary data can be found online at: www.aginganddisease.org/EN/10.14336/AD.2022.0218.


