# Supplementary material for: UPP1 Promotes Lung Adenocarcinoma Progression through Epigenetic Regulation of Glycolysis
Source: Aging Dis. 2022 Oct 1;13(5):1488–503. doi: 10.14336/AD.2022.0218 (PMC9466982; doi:10.14336/AD.2022.0218)
Supplement: Supplementary file 1 [file AD-13-5-1488-s.pdf]

SUPPLEMENTARY DATA

UPP1 Promotes Lung Adenocarcinoma Progression through Epigenetic Regulation of Glycolysis

Xuan Wang[1,#,*], Zheng Wang[2,#], Renhong Huang[2,#], Zhouyi Lu[1], Xiaofeng Chen[1*], Dayu Huang[1*]

© 2022. Wang X. et al. Published online at http://www.aginganddisease.org/EN/10.14336/AD.2022.0218

SUPPLEMENTARY DATA

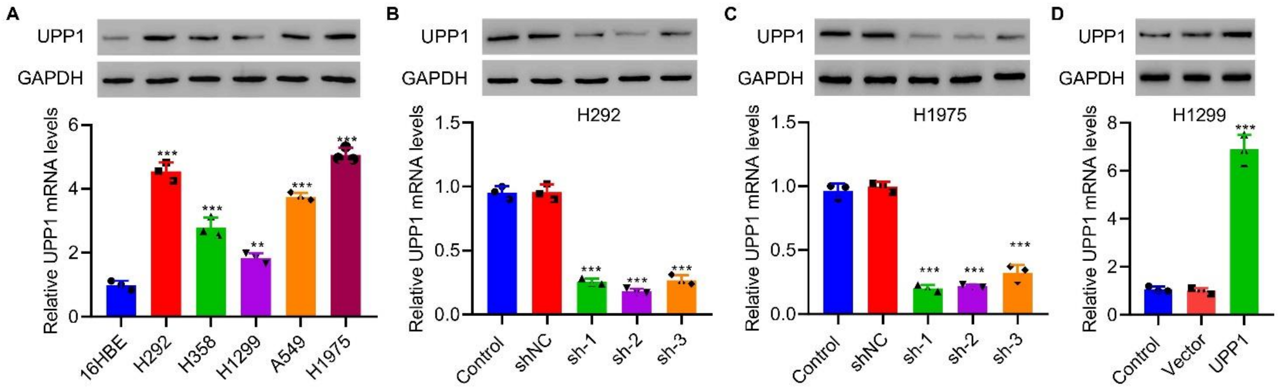

Supplementary Figure 1. (A) Real–time PCR and western blot analysis of UPP1 expression in LUAD cell lines and 16HBE, a human bronchial epithelial cell line. (B-C) Stable knockdown of UPP1 in H292 and H1975 cells infected with lentiviral sh-1, sh-2 and sh-3 sequences. The knockdown effect was verified at the mRNA and protein levels. (D) Stable overexpression of UPP1 in H1299 cells by lentiviral UPP1 overexpression sequences. The overexpression effects were verified at the mRNA and protein levels. One-way ANOVA in (Fig. S1A-D). The experiments were independently conducted three times. ***P<0.001 vs. 16HBE, shNC, or vector.

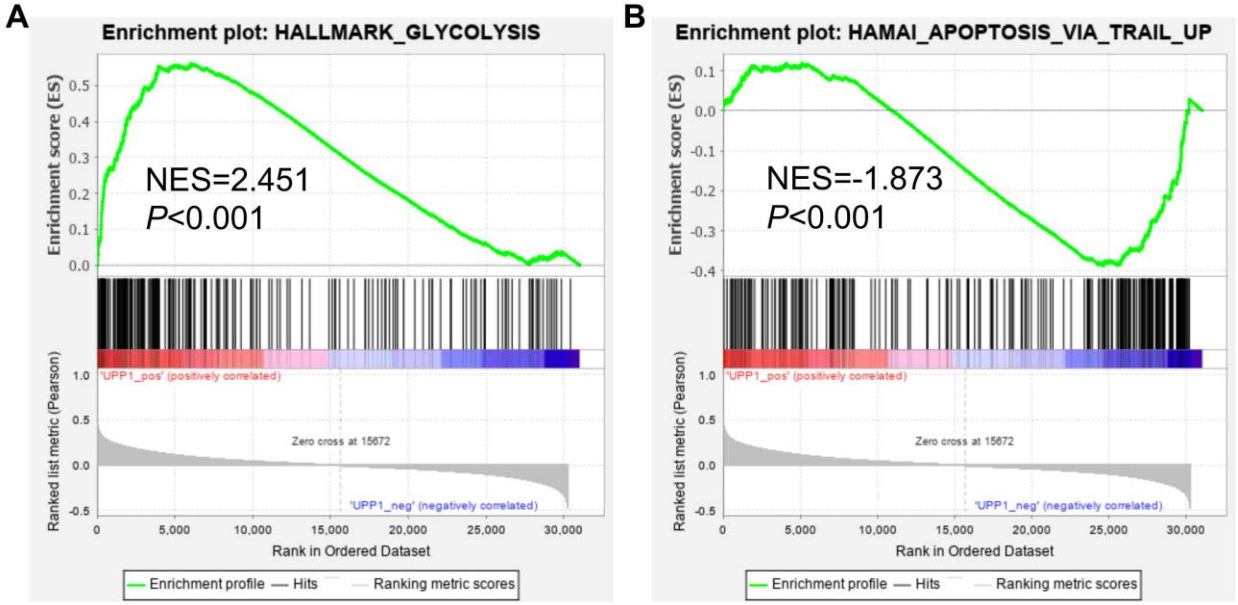

Supplementary Figure 2. GSEA showed that the expression of UPP1 is related to tumor glycolysis (A) and the apoptosis (B) pathway. Scale bar: 100 μm

© 2022. Wang X. et al. Published online at http://www.aginganddisease.org/EN/10.14336/AD.2022.0218

SUPPLEMENTARY DATA

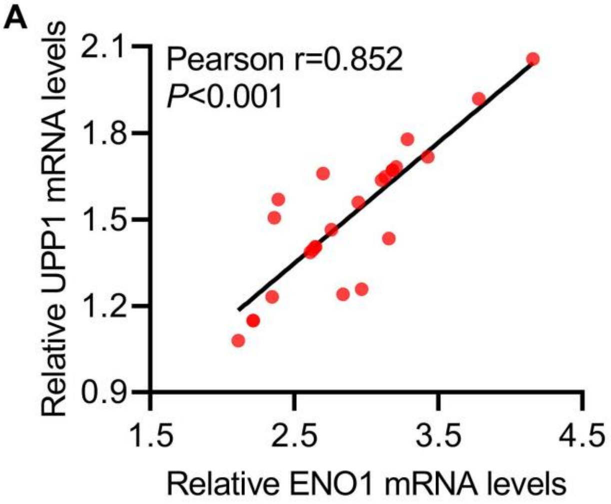

Supplementary Figure 3. UPP1 mRNA expression was positively correlated with the expression of ENO1 (A) and LDAH (B) in LUAD patients in our hospital cohort (n=25).

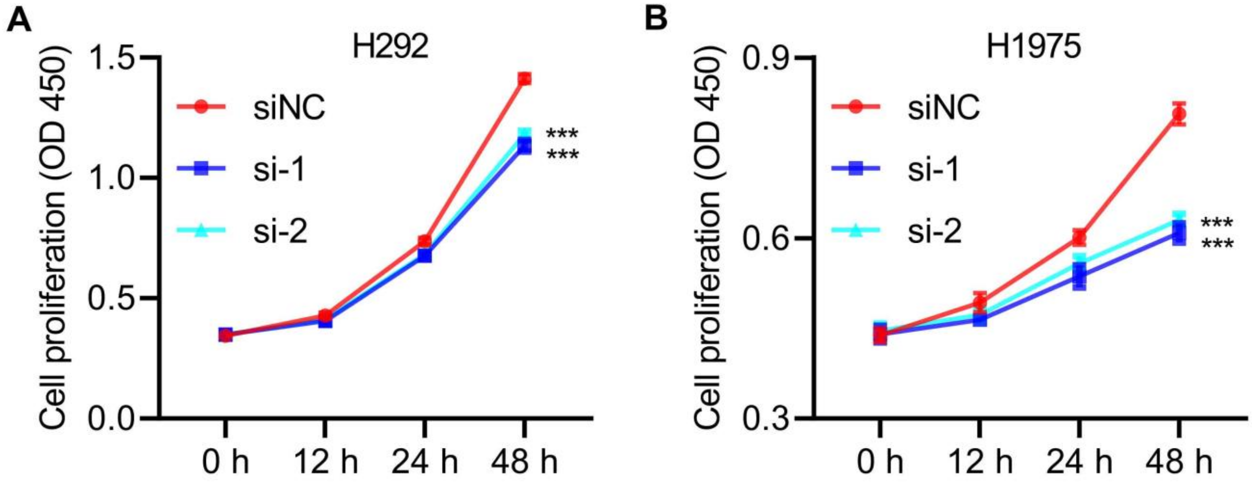

Supplementary Figure 4. Knockdown of UPP1 suppresses tumor progression in vivo. Cell proliferation was determined at 0h, 12h, 24h and 48h by the absorbance value (OD) at 450 nm using a microplate reader after Knockdown of UPP1 by siRNA in H292 (A) and H1975 (B) cell lines. One-way ANOVA in (Fig. S4A-B). **P<0.01, ***P<0.001

© 2022. Wang X. et al. Published online at http://www.aginganddisease.org/EN/10.14336/AD.2022.0218

SUPPLEMENTARY DATA

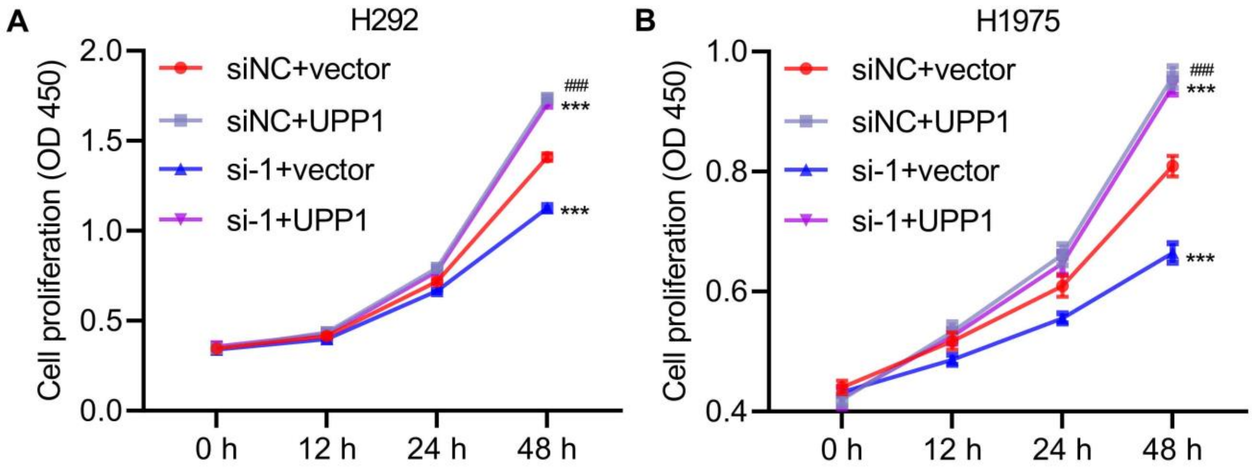

Supplementary Figure 5.. UPP1 knockdown could be rescued UPP1-overexpressed plasmid transfection. Cell proliferation was determined at 0h, 12h, 24h and 48h by the absorbance value (OD) at 450 nm using a microplate reader of the control group (siNC+vector), the UPP1 knockdown group by siRNA intereference group (si1+vector), the UPP1-overexpressed group by plasmid transfection group (siNC+UPP1) and the UPP1 knockdown group by siRNA intereference plus UPP1-overexpressed plasmid transfection group (si-1+UPP1) in H292 (A) and H1975 (B) cell lines. One-way ANOVA in (Fig. S5A-B). ***P<0.001 vs. siNC+vector. ###P<0.001 vs. si-1+vector.

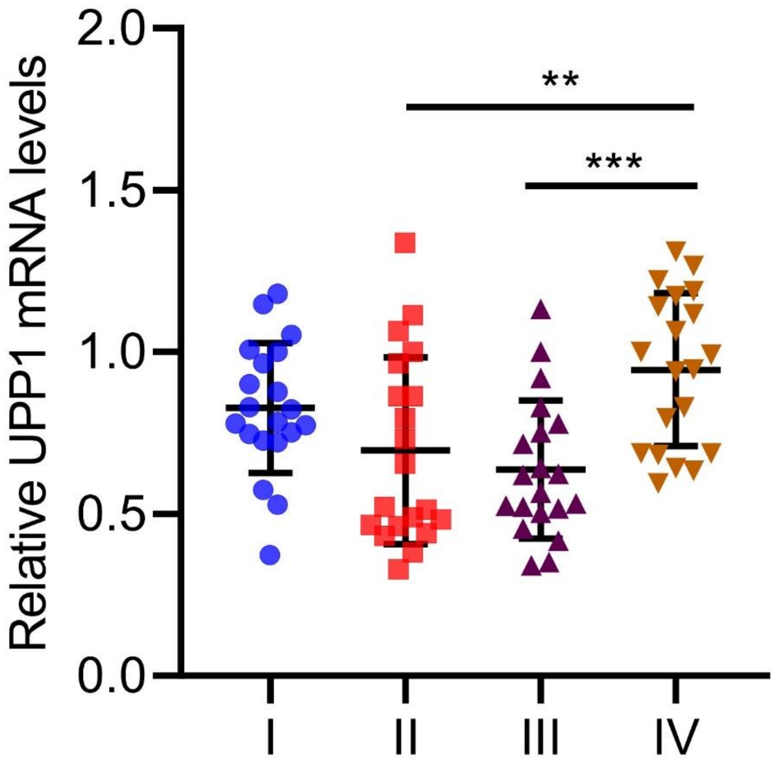

Supplementary Figure 6. The expression of UPP1 in different stage of LUAD patients. qRT-PCR was applied to detect the expression of UPP1 mRNA in stage I (N=24), stage II(N=23), stage III(N=20) and stage IV of LUAD patients (N=20). One-way ANOVA in the figure.

© 2022. Wang X. et al. Published online at http://www.aginganddisease.org/EN/10.14336/AD.2022.0218

SUPPLEMENTARY DATA

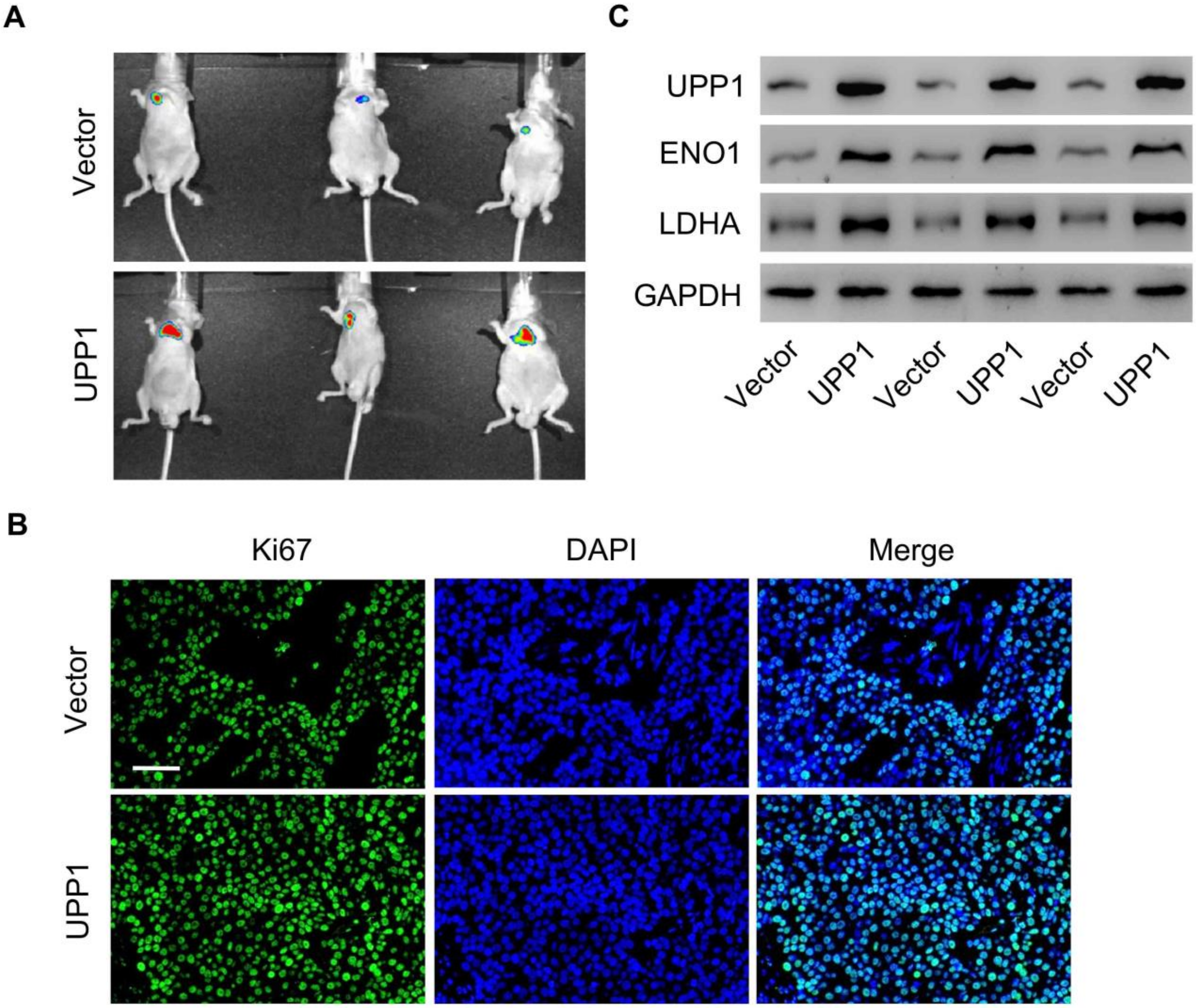

Supplementary Figure 7. UPP1 drives glycolytic metabolism in vivo. (A) UPP1 overexpression promotes subcutaneous tumor growth in nude mice (Vector group, n=3; UPP1 group, n=3). (B) UPP1 overexpression increases the protein expression of ENO1 and LDHA. (C) UPP1 overexpression promotes proliferation of H1975 cells.

© 2022. Wang X. et al. Published online at http://www.aginganddisease.org/EN/10.14336/AD.2022.0218

SUPPLEMENTARY DATA

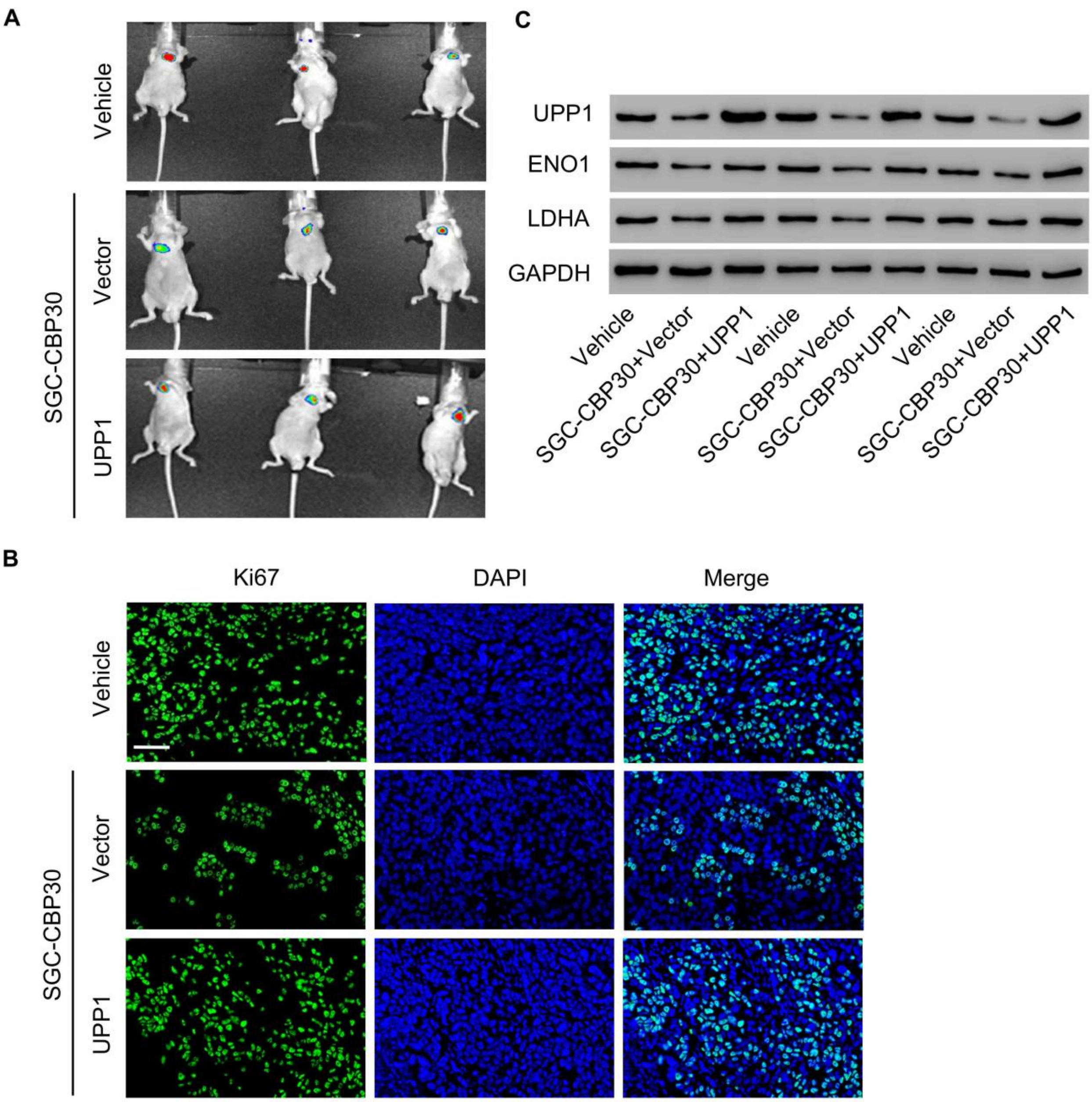

Supplementary Figure 8. UPP1 overexpression inhibits the antitumor effect induced by SGC-CBP30 in vivo. (A) SGC-CBP30 inhibited subcutaneous tumor growth in nude mice, while UPP1 overexpression restored the effect (Vehicle, n=3; Vector, n=3; UPP1, n=3). (B) SGC-CBP30 inhibit cell proliferation, while UPP1 overexpression restored the effect. (C) The protein expression of ENO1 and LDHA of subcutaneous tumor was reduced in the SGC-CBP30 group, but UPP1 overexpression increased their expression.

© 2022. Wang X. et al. Published online at http://www.aginganddisease.org/EN/10.14336/AD.2022.0218

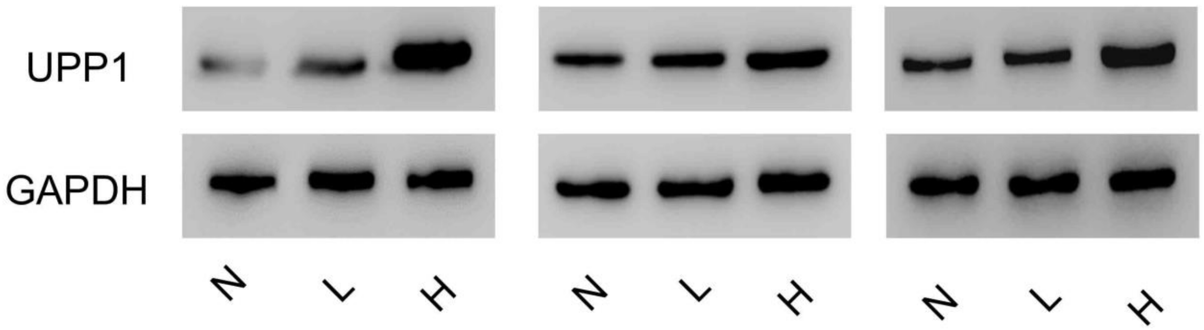

Supplementary Figure 9. UPP1 was higher expressed in tumors. Western blot was applied to test the expression of UPP1 in normal tissues, UPP1 mRNA low expression tumors and UPP1 mRNA high expression tumors.

© 2022. Wang X. et al. Published online at http://www.aginganddisease.org/EN/10.14336/AD.2022.0218
